# A narrative review of MRI changes correlated to signs and symptoms of autism

**DOI:** 10.1097/MD.0000000000030059

**Published:** 2022-08-26

**Authors:** Nahla L. Faizo

**Affiliations:** Radiological Sciences Department, College of Applied Medical Sciences, Taif University, KSA.

**Keywords:** Autism spectrum disorder, brain, communication, MRI, neuroimaging, repetitive behavior

## Abstract

Autism spectrum disorder (ASD) is a neurodevelopmental disorder that occurs during early childhood. The change from being normal across several contexts to displaying the behavioral phenotype of ASD occurs in infants and toddlers with autism. Findings provided by magnetic resonance imaging (MRI)-based research owing to the developmental phase, including potential pathways underlying the pathogenesis of the condition and the potential for signs and symptomatic risk prediction. The present study focuses on the characteristic features of magnetic resonance imaging autistic brain, how these changes are correlated to autism signs and symptoms and the implications of MRI as a potential tool for the early diagnosis of ASD.

PRISMA style was used to conduct this review. Research articles related to the key concepts of this review, which is looking at MRI brain changes in autistic patients, were revised and incorporated with what is known with the pathophysiology of brain regions in relation to signs and symptoms of autism.

Studies on brain MRI of autism were revied for major brain features and regions such as brain volume, cortex and hippocampus. This review reveals that brain changes seen in MRI are highly correlated with the signs and symptoms of autism.

There are numerous distinct features noted in an autistic brain using MRI. Based on these findings, various developmental brain paths and autistic behavior culminate in a typical diagnosis, and it is possible that addressing these trajectories would improve the accuracy in which children are detected and provide the necessary treatment.

## 1. Introduction

Autism spectrum disorder (ASD) is a neurodevelopmental impairment characterized by chronic social contact difficulties and limited repetitive conduct.^[1]^ Qualitative deficiencies in social contact and stereotyped forms of actions or desires are the 2 major attributes of ASD.^[2]^ A deeper perception of the condition and how it manifests in a particular subject will contribute to more successful action strategies to meet the individual’s medical needs.

A comparatively small developmental timeframe in which ASD unfolds presents researchers with the ability to differentiate developmental events prior to its onset. Interventions to understand how early in childhood the condition emerged are primarily based on observational approaches or inferences based on recently diagnosed children’s observations and propositions based on results from older children or adults. In their ability to capture the specific and sometimes nuanced occurrences that accompanied a diagnosis, these techniques advanced awareness but were eventually limited.^[3]^ With respect to the brain, retrospective approaches have largely been restricted to head circumference records.

The etiology of ASD is not well understood. In addition, neurobiological observations that have been documented in ASD change with age. For instance, head size, which has been reported to be smaller at birth in individuals with ASD, quickly develops to be greater than that in normal infants and then normalizes in maturity.^[4]^ Magnetic resonance imaging (MRI) is one of the main radiological procedures that help in explaining the diverse ways in which the brain develops, recognize features of brain growth that diverge from the standard evolutionary trajectory, and document brain structure, activity, and metabolism characteristics. It is the only high-resolution, comprehensive technique appropriate for use in children in the clinical testing environment as a noninvasive instrument.

Although Autism has core features of the disease, it is heterogenous with a wide variety of signs and symptoms. Thus, establishing a database of an MRI biomarkers for Autism is considered a crucial phase for more detailed interpretation of the diagnosis which will lead to a better-targeted and tailored action strategy to meet the medical needs of those patients. The brain functions of each individual include several separate sets of networks or circuits. These loops are composed of scattered regions of gray matter (GM) entangled by individual tracts and paths of white matter (WM). Both parts of the system must be preserved for efficient operation of circuits. There are various possible causes of improper circuit function, including the arrangement of the processing units in the GM, connectivity of the dispersed elements of the circuit, and uneven inputs to the circuit.^[5]^

The present review article summarizes the autistic brain MRI changes and the implications of MRI as a potential tool for the early diagnosis of ASD. In addition, it reviews how these changes are correlated to the signs and symptoms of autism. Furthermore, it goes through the correlation of these MRI findings with the management strategies for autism.

## 2. Search methodology

With respect to the dramatic development of brain MRI in explaining the pathophysiology and structural changes in autism, this narrative review was conducted using Preferred Reporting Items for Systematic Reviews and Meta-Analyses (PRISMA) style. The purpose of this review was to provide a gathered and well-defined information about the MR brain changes in autistic patients and incorporated it with what is known regarding the pathophysiology of these brain regions in relation to signs and symptoms of autism.

By searching PubMed for articles related to the key concepts of this review which includes autism, MRI brain changes and signs and symptoms of autistic disorders using the exact words or their synonyms, many publications were founded. Upon reviewing the related literature, the author selected research articles fully published in English. Comprehensive and relevant information on autistic brain changes using MRI were extracted and incorporated with what is known with the pathophysiology of brain regions in relation to signs and symptoms of autism. There were no date restrictions were used in order to gather the most relevant data using different MRI protocols of structural and the advanced structural imaging. Ethical approval was not necessary, as this study does not involve patient consent nor patients’ new information.

### 2.1. MRI features of the brain in autistic patients

Since their introduction in the 1980s, MRI techniques have evolved as an efficient approach that enables noninvasive clinical diagnosis of different diseases and anomalies. MRI experiments have shown several explanations of ASD-based neurodevelopmental traits, as it is an established reality that neuroimaging is one of the few tools that enables direct in vivo brain analysis.^[6]^ To better explain autism, many MRI modalities, such as sMRI and fMRI, have been used to analyze brain structures and networks related to ASD (Table 1).^[7–14]^Structural MRI (sMRI) studies have reported different outcomes in the preceding years, however, there are anomalies in GM and WM with those of regional brain discrepancies in the ASD and control groups.^[15]^ Here, a summarized revision on autism brain morphology is discussed including brain volume, cortical areas, and regional brain structures (amygdale, hippocampus, and posterior crainal fossa).

**Table 1:** Alterations in brain structures in children with ASD along with their associated functional symptoms.

Brain structure	MRI structural finding	MRI functional changes
Hippocampus^[8]^	Enlarged	Disruptive activity during repetitive behavior
Amygdala^[9]^	Sizes of the right and left amygdala were increased as compared to the total brain volume	Social interaction and communication deficits
Cerebellum^[10]^	Enlarged	Motor deficits and impairment in cognitive functions such as associative learning, verbal ability, planning, and working memory
Frontal cortex^[12]^	Abnormal growth patterns with precocious overgrowth	Impairments in social interaction and emotion
Total brain volume^[13]^	Increased	Atypical developmental issues
Striatum^[14]^	Volume variation	Repetitive or restricted behavior

#### 2.1.1. Brain volume.

Some studies examined the brain volume of ASD patients using MRI and revealed atypical developmental patterns of enlarged brain volume in the younger group with ASD, while a diminished or no variation in volume compared to typically developing control was observed in the older ASD population.^[16]^ The average brain volume was noted to be increased in infants aged 18–35-month-old, and their normal birth head size appeared to be slightly greater at 12 months of age relative to the control group.^[17]^ Consistent with the above reports, similar results were observed for autistic individuals in the age group of 10 years.^[18]^

During early childhood in ASD, development of the brain appears to be primarily responsible for an expanded frontal and temporal lobe brain volume accompanied by halted growth and a potential decrease in brain volumetric capability after an approximate age of 10 to 15 years.^[15]^

It has been shown that ASD develops extraaxial fluid, which is distinguished at 6–9 months of age by elevated cerebral spinal fluid (CSF) over the frontal lobes, which occurs at 12–15 and 18–24 months as well.^[19]^ This contributes to relatively large cerebral volumes, which appear to rise in males as compared to females at an increased age. This observation, however, contradicts the prior report by Schumann et al, which indicated a higher enlargement in females.^[20]^ Sparks et al and Akshoomoff et al study also revealed enhanced cortical volumes in autistic children aged approximately 4 years and 6 years, respectively.^[21]^ In the latter study, elevated total cerebral volume and raised levels of WM and GM volumes were identified in children with both low-functioning and high-functioning autism, with substantially greater total cerebral volume in the low-functioning group.^[21,22]^

Further, another study on 4-year-old male children focused on obtaining features from GM, WM, and CSF to identify autistic brains. The results revealed that only GM functionality implied a classification performance that exceeded 80% in multiple subregions.^[23]^ A research study led by Bonilha et al showed an improvement in the GM volume in the parietal lobes, medial and dorsolateral frontal regions, and lateral and medial portions of the temporal lobes in autistic males at approximately of 13 years, and a noticeable decline in the volume of WM in the frontal, parietal, temporal, and occipital lobes.^[24]^

#### 2.1.2. Cortical area.

The focus of numerous sMRI research findings on ASD has been the uppermost brain layer leading to human intelligence and perception. Atypical cortical folding is one of the core characteristics observed in the brains of individuals with ASD.^[25]^ The mechanical strain of axonal WM fibers pulling stress on the neocortex might be an underlying cause of irregularities in cortical folding.^[26]^ These data show that in patients with ASD, there is an unusually enhanced frontal lobe gyrification. In early adolescence and adulthood, regional cortical folding is enhanced in the bilateral posterior brain regions. On the other hand, it has been reported elsewhere that in the right inferior frontal and medial parieto-occipital cortices, decreased local gyrification has been identified in children with ASD, while it is in the left supramarginal gyrus for patients aged 8 to 40 years.^[27]^ These distinct results indicate that the basic pattern of cortical gyrification changes over the lifetime, and the facets of cortical geometry are highly affected by genetic and environmental influences. Dierker et al observed substantial bilateral variations in sulcal depth in the small anterior insula, frontal operculum, and temporoparietal junction portions.^[28]^

Two consecutive longitudinal experiments on the same group of participants at a mean age of 17.4 and 19 years, respectively, led by Wallace et al revealed progressive cortical thinning in the autistic brain with reference to controls in 2 regions of the left hemisphere, including the posterior part of the ventral temporal cortex and the superior parietal cortex.^[29]^

#### 2.1.3. Regional brain structures.

The rapid growth of the GM cortical surface region in ASD tends to be linked to disrupted cortical WM maturation. The mediation of clinical phenotypes of ASD usually involves the frontotemporal lobe, frontoparietal cortex, amygdala, hippocampus, basal ganglia, and anterior cingulate cortex.^[30]^ For instance, anomalies in the inferior frontal gyrus, superior temporal sulcus, and Wernicke area can be associated with deficits in the development of natural language and social focus. In contrast, it has been reported that the frontal lobe, superior temporal cortex, parietal cortex, and amygdala may instigate social interaction impairments.^[31]^ Outcomes from a vertex-based study analysis showed that the population with ASD appeared to have age-related influences on thinner cortices and decreased surface area. These results indicate that a cortical growth plot is curvilinear over the human lifetime, and there is an indication of irregular cortical widening at an early life accompanied by accelerated adult cortical thinning in adulthood.^[32]^

##### 2.1.3.1. Amygdalae.

The amygdalae widen substantially to the average increase in the whole cerebral volume in autistic subjects. Nordahl et al reported that amygdalar volume magnification was observed in subjects aged 37 months and after 1 year of follow-up, but with a higher incidence for the latter category, suggesting that the observed magnification occurs at an early age of about 3 years.^[33]^ In addition, the same study revealed that in ASD, the volume of the amygdala and the microstructure of the links between the amygdala and cortex are altered.

##### 2.1.3.2. Hippocampus.

In autistic children aged 3–4 years, inflated hippocampi were observed, although another study documented an increase in size only in the right hippocampus in children aged approximately 10 years.^[22–34]^ Compared with controls and parents of autistic adolescents, the hippocampus of autistic adults was substantially enlarged, as reported by Rojas et al In contrast to the controls, the left hippocampus was greater in the parents of autistic children, as well as those of autistic adults.^[35]^ Hence, it can be inferred that the hippocampus is enlarged in autistic patients of all age groups.

##### 2.1.3.3. Posterior cranial fossa.

With ASD from an early age of approximately 2 years following the subsequent life periods, the posterior fossa constructs are greatly affected. Bonilha et al reported an improvement in GM volume in the cerebellum in children with autism. Autistic children and adolescents’ cerebellar volumes were aligned with those of adults. McAlonan et al recorded reduced GM density in the frontostriatal and cerebellar regions together with extensive WM variations, and lower brain stem and overall brain stem GM concentrations were also observed in autistic adults.^[36]^ At such an early age, cerebral modifications in the autistic brain entail age-proportional widening of the lobes and increases in prefrontal WM and GM areas. In early childhood, the increasing cerebral volume, namely GM and WM, suggests a decreased density of GM and WM at the same time. It is also noted from relevant studies that children with autism also undergo major differences in cortical thickness.

### 2.2. Correlation between MRI brain findings and signs and symptoms in autistic patients

The more often observed signs and symptoms in autistic patients are confined to repetitive or restricted behavior and aberrant interaction in the social sphere. The following section describes the correlation of the stated behaviors in autistic patients with atypical brain functions based on MRI findings.

#### 2.2.1. Repetitive or restricted behavior.

A significant diagnostic criterion for ASD is the occurrence of repetitive or restricted behavior (RRB), which is related to striatal variations. These noted behaviors are noticeable in infants with ASD, and even continue in children with ASD. Functional magnetic resonance imaging (fMRI) has proved to be a helpful instrument for studying disordered neurobiological activity in ASDs as it has a good spatial resolution, to encode metabolic measures of neuronal activity related to relevant neurocognitive functions, fMRI uses advanced pulse sequences.^[37]^ Goldberg et al also explored the neuronal basis of error monitoring using fMRI in children with ASD. Compared to the children in the control group, children with ASD displayed elevated activation in the anterior medial prefrontal cortex and left superior temporal gyrus. These observations related to activation in the anterior medial prefrontal cortex and left superior temporal gyrus often led to a change in the emotional state of patients with ASD. It ends up being more flexible and reactive to contingencies that are more static and repetitive in nature. A change in the internal state (emotional state) has been reported, which leads to errors in children with ASD.^[38]^ Another group of researchers examined 14 control and ten ASD participants to determine whether the structure and function of the anterior cingulate cortex (ACC) during response monitoring is correlated with repetitive activity in ASD.^[39]^ The microstructural integrity of the WM underpinning brain area using diffusion tensor imaging measures of fractional anisotropy (FA) was analyzed in a group of 12 subjects in the control group and 12 adult ASD participants, since it is the result of synchronized operation in ACC circuits. It was interpreted from the present study that, compared to individuals in control groups, ASD individuals rendered several antisaccade errors and reacted more rapidly in trials, displayed decreased discrimination in the rostral ACC (rACC) between error and correct responses, exceptionally elevated activation in correct trials, and decreased FA in WM underlying ACC. These results indicate that functional and structural abnormalities in the ACC can lead to repeated abnormal behaviors in ASD. It is assumed that the rACC behavior after errors represents the aversive evaluation of the noted error. FMRI was employed to investigate the functional variations surrounding RRBs in children with ASD.^[40]^ In this study, children with ASD showed delayed activity connected to visuomotor sequence learning in brain regions such as the posterior cingulate cortex. They proposed that variations in brain processes may facilitate basic sequence learning in ASD and help understand the behavioral manifestations of impairments associated with ASD in ability development. The symptoms of RRBs normally do not appear in the same manner and are modified over time in different ways. Multiple studies have stated that, based on age, RRBs present differently.^[41]^ It was found that children with ASD aged 18 months to 24 months displayed a higher incidence and longer length of RRBs. However, the same findings have not been reported in older subjects in another research study.^[42]^ More motor and sensory repetitive behaviors were exhibited by younger ASD children, whereas older children with ASD had more complicated behavioral patterns.^[43]^ It has also been suggested that irregular sensory perception is related to RRBs in ASD. Clery et al analyzed brain functions consisting of constant visual moving stimuli during task execution. In the bilateral occipital cortex and in the ACC, adults with ASD displayed enhanced activity correlated with reduced activation compared to control groups in the superior and middle frontal gyri. In ASD brains, atypical coordination was also established between the frontal and occipital regions.^[44]^

#### 2.2.2. Aberrant interaction in the social sphere.

Children with ASD usually display reduced language regulation, which causes delays in social communication. Neurobiological variations in delayed language production between children with ASD and the control group have been explored in several fMRI studies.^[45]^ The neurobiological deficiencies in recognizing ambiguity in high-functioning children with ASD were investigated by Wang et al using fMRI. In comparison to earlier research suggesting hypo-activation of domains engaged in interpreting others’ mental states, children with ASD demonstrated hyper-activation in both the right inferior frontal gyrus (IFG) and bilateral temporal regions as opposed to those in the control group.^[46]^ Urbain et al have demonstrated a strong association between ACC hypoactivation and enhanced lack of social contact in children with ASD. They proposed that the ACC plays a crucial role in the management of both cognitive and emotional processing.^[47]^ The lack of the ability to visually examine emotional facial expressions, the subsequent internal duplication through the mirror neuron system, and the ability to communicate it to the limbic system and control the emotion conveyed may be a possible cause for deficits in the social recognition of children with ASD, as evidenced in another study.^[48]^

There is a heterogeneous spectrum of language acquisition impairments in ASD, from the loss of comprehension to high-order communication, such as functional language deficits. Many neuroimaging studies have shown that anomalous activations in the Broca and Wernicke regions can play a pivotal role in the representation of disabled language in ASD.^[49]^

### 2.3. Management strategies for autism

There is no specific or absolute treatment for ASD. However, there are ways in which signs can be managed, and knowledge is maximized. When ASD patients undergo effective therapy and treatment, they have the greatest chance of using any of their competencies and abilities. For each patient, the most successful treatment and strategy were always different. However, many individuals with ASD adapt better to highly organized and advanced programs.^[50]^ Therapy may allow people with autism to function normally in certain cases. Evidence suggests that early diagnosis and treatment are most likely to have remarkable beneficial effects on symptoms and later abilities, such as during preschool or before. Because signs can vary with ASD and other conditions, such as attention deficit hyperactivity disorder, it is important to identify care on a person’s individual needs rather than the diagnosis mark.^[51]^

The major treatment modalities include behavioral management therapy, cognitive behavior therapy, early intervention, educational and school-based therapies, joint attention therapy, medication treatment, nutritional therapy, occupational therapy, parent-mediated therapy, physical therapy, social skills training, and speech-language therapy.

ASD involves a cascade of events, and while this cascade of results appears over a larger temporal duration, ASD occurs within a particular brain growth window marked by significant improvements in both rate and scale. During this brief but critical phase of development, an infant progress from being normal to manifesting disability and developmental delays across various cognitive and behavioral spheres. Brain imaging studies, along with prospective developmental trials in infants and toddlers, have greatly improved our understanding of early autism growth and possible causes of susceptibility to these conditions.^[52]^ MRI analysis has been routinely utilized in such a pursuit, and based on the pathophysiological findings, autism is better diagnosed at an early stage of childhood. There are numerous distinct features noted in an autistic brain that differentiate it from the normal brain (discussed earlier). Based on these findings, clinicians can opt for suitable treatment options specific to the individual. Although multiple treatment options are available, it can be stated that there is no “magic” treatment bullet available for all individuals with autism. It is pertinent to state that no single, possibly the best approach to autism exists. Instead, various developmental paths culminate in a typical diagnosis, and it is possible that addressing these trajectories would improve the accuracy in which children are detected and provide the necessary treatment.

## 3. Conclusion

A substantial rise in awareness linked to the neurodevelopment of ASD has been characterized in the last 2 decades. This has generated fresh perspectives into improvements in the development and connectivity of the brain that occur due to clinical symptom convergence. To properly define the early neurodevelopment of ASD, 1 key factor is to promote attempts to include targeted intervention or prevention. By optimizing long-term cognitive and behavioral effects and substantially reducing lifetime costs aligned with treatment, early intervention can be particularly successful in strengthening the influence of ASD. However, despite such action, only a handful of young children with ASD make substantial improvements, and many demonstrate little to no change in key deficit domains. The noted pattern may be due to a lack of accuracy in the way treatments are adapted to human needs. The traditional trial-and-error strategy to determine successful intervention threatens the waste of precious time and energy since early childhood provides a limited window of substantial brain and behavioral permeability. The solution could be an intervention strategy that considers heterogeneity by personalization. Cognitive and behavioral variables such as cognitive skill pretreatment or functional communication capacity may present some clarification on the form, length, and severity of the action, but there is little codified influence of these variables alone. Compensating for variations in the neural phenotype of ASD may strengthen further interactions between a particular child’s unique needs and therapeutic format, intensity, and duration. To do this, innovative and developmentally aware interventions are needed to exploit behavioral and neurobiological heterogeneity to guide risk identification and treatment personalization. While these findings have summarized anomalous patterns of brain activity in ASD individuals, studies focused on identifying modes of action of therapeutic response in ASD have seldom extended these frameworks to longitudinal treatment outcome studies. Functional brain imaging will help to enhance our perception of the pathophysiology of ASDs as neuroimaging and data exchange methods advance, with the eventual aim of enhancing ASD diagnosis and care.

## Author contributions

This is a single author review paper by me (Nahla L. Faizo). I am responsible the conception, design/methodology, analysis, interpretation of data, writing, reviewing and editing the manuscript.
